# Seed-mediated synthesis of monodisperse plasmonic magnesium nanoparticles†

**DOI:** 10.1039/d3cc00958k

**Published:** 2023-04-12

**Authors:** Vladimir Lomonosov, Elizabeth R. Hopper, Emilie Ringe

**Affiliations:** a Department of Materials Science and Metallurgy, University of Cambridge 27 Charles Babbage Road Cambridge CB3 0FS UK er407@cam.ac.uk; b Department of Earth Sciences, University of Cambridge Downing Street Cambridge CB2 3EQ UK; c Department of Chemical Engineering and Biotechnology, University of Cambridge Philippa Fawcett Drive Cambridge CB3 0AS UK

## Abstract

We reduce di-*n*-butylmagnesium with arene (naphthalene, biphenyl, phenanthrene) radical anions and dianions to obtain metallic, plasmonic Mg nanoparticles. Their size and shape depends on the dianion concentration and reduction potential. Based on these results, we demonstrate a seeded growth Mg nanoparticle synthesis and report homogeneous shapes with controllable monodisperse size distributions.

## Abstract 2

We demonstrate the seed-mediated synthesis of plasmonic, metallic magnesium nanoparticles, leading to controllable and monodisperse nanoparticle size distributions.

## Graphical abstract

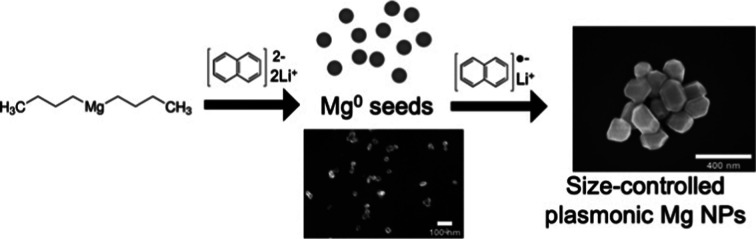

Nanoparticles (NPs) of some free-electron metals sustain light-driven resonant oscillations of their free electrons called localised surface plasmon resonances (LSPRs). At the resonant frequency, the extinction cross-section of a plasmonic NP can exceed its physical cross-section, resulting in light antenna with wavelength-dependent scattering, absorption, and strong local electric field enhancement. These effects underpin LSPRs’ applications in photothermal cancer therapy,^1^ photocatalysis,^2–4^ sensing^5^ and surface-enhanced spectroscopies.^6,7^

The typical plasmonic metals, Ag and Au, have been challenged in the past decades by earth-abundant alternatives such as Cu^8^ and Al.^9^ Recently, Mg has attracted attention due to its plasmonic response across the ultraviolet, visible and near-infrared,^10–13^ in addition to its earth abundance and biocompatibility. This has already led to Mg being explored as an alternative plasmonic material for sensing,^14^ cancer therapy,^15,16^ and photocatalysis.^17^ However, Mg is more than a replacement for Ag and Au: it is different. Its resonant range is broad, and Mg's hexagonal lattice leads to NP shapes strikingly distinct from those of all other common plasmonic elements (Al, Ag, Cu, Au), which adopt a face centred cubic (FCC) lattice.

Efficient utilisation of plasmonic properties relies on tuning the LSP frequency, usually achieved by tuning the NP's size and shape. For Mg, an early report of the reduction of MgCl_2_ to a “powder” by Rieke *et al.* in the presence of an arene (*e.g.*, naphthalene or biphenyl)^18^ inspired a recent systematic study^19^ in which we varied synthetic parameters including the reducing agent's potential (by selecting the arene) to obtain metallic Mg NPs from di-*n*-butylmagnesium. The mean NP size could be tuned between 80 and 1300 nm, however, the overlap between the reaction's nucleation and growth stages led to significant size and shape heterogeneity, the latter due to the ability of Mg to twin along multiple planes leading to various rod shapes^20^ in addition to single crystalline hexagonal platelets.^10^

Seed-mediated approaches, where seeds are formed in reaction conditions different than that of the growth phase, have been key to reducing size and shape heterogeneity in many NP syntheses.^21,22^ However, this method remains elusive for the reactive plasmonic metals Al^23^ and Mg^11^ due to the spontaneous formation of a surface oxide during the intermediate reaction workup. Here we demonstrate a one-pot, two-step process in which a highly reductive arene dianion is used for the nucleation step, then converted *in situ* to the radical anion for the growth phase. This flexible synthetic platform yields drastic improvements of the shape dispersion, previously unreported shapes, and the ability to control NP size.

A typical^10,19,20^ single step synthesis of Mg NPs was performed by sonicating a 1 : 1 molar ratio of naphthalene and Li pellets in anhydrous THF, then injecting di-*n*-butylmagnesium into the LiNapht solution (Fig. 1a) such that the arene radical anion was in slight stoichiometric excess of the Mg precursor. The reaction proceeded for 18 h, and the products were recovered after quenching and centrifugation. The resulting platelets and folded platelets are faceted, large and heterogeneous (mean ± standard deviation 900 ± 200 nm) as shown in the scanning electron microscopy (SEM) images in Fig. 1b and Fig. S1, ESI.† Their plasmonic, metallic character and the thin self-limiting oxide layer has been reported previously.^10,13,20,24,25^

**Fig. 1:**
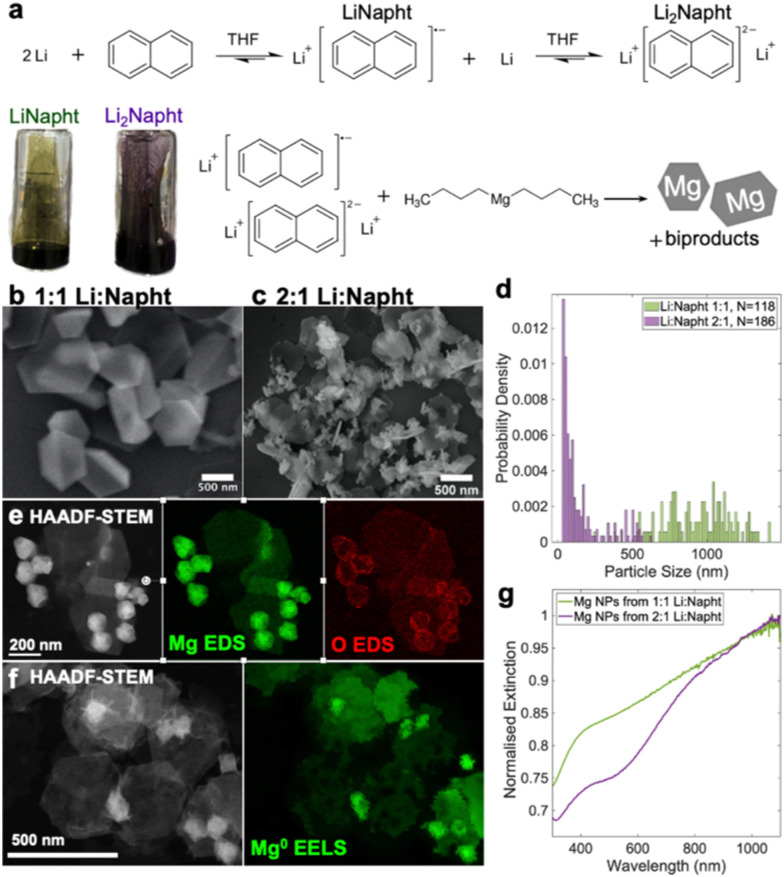
Synthesis of Mg NPs using Li_2_Napht and LiNapht as reducing agents. (a) Reaction scheme for the formation of LiNapht and Li_2_Napht from Li and naphthalene in THF, their respective colour, and their reaction with di-*n*-butylmagnesium; representative SEM and size distributions of Mg NPs synthesised with (b and d) 1 : 1 and (c and d) 2 : 1 Li:naphthalene; (e and f) HAADF-STEM images, (e) STEM-EDS map of Mg and O, and (f) STEM-EELS maps of the bulk plasmon of metallic Mg for Mg NPs synthesised using 2 : 1 Li:naphthalene; (g) optical extinction spectra of Mg NPs dispersed in isopropanol.

Changing the Li:naphthalene ratio from 1 : 1 to 2 : 1 modified the colour of the solution from dark green to purple (Fig. 1a). This is due to the formation of a dianion of naphthalene (Li_2_Napht), as obtained by Smid when excess Li reacts with naphthalene in THF.^26^ This highly reduced species has a reduction potential 0.2–0.3 eV more negative than the radical anion,^27^ and substantially impacts the formation of organoLi compounds with catalytic amounts of arene.^28^ However, the role of the dianion in Mg NP synthesis has not been previously described. Other Mg syntheses with Li in excess have been reported^16,18,29–31^ where Li and the arene are mixed in the presence of the Mg precursor, precluding dianion formation. A recent report claims that the sonication time of Li and naphthalene changed LiNapht's reduction potential and thus the final product.^16^ We believe this is a misinterpretation, as the LiNapht reduction potential is essentially constant. Here we show systematically that the reaction outcome depends on the radical anion to dianion ratio.

The presence of a dianion significantly impacted the size and shape of the NPs produced. Mg NPs obtained after the addition of di-*n*-butylmagnesium to the 2 : 1 Li:naphthalene solution had a bimodal size distribution, with thin hexagonal platelets as the larger NP size population of 320 ± 170 nm, and small, thick NPs with sizes of 54 ± 18 nm (Fig. 1c and d and Fig. S1, ESI†). Both types of NPs are composed of a thin oxide layer, evidenced by scanning transmission EM energy dispersive X-ray spectroscopy (STEM-EDS, Fig. 1e), atop a metallic Mg core, confirmed by the bulk plasmon peak visible in STEM electron energy loss spectroscopy (STEM-EELS, Fig. 1f and Fig. S2, ESI†), in agreement with our previous results.^10,19,20,24,25^ The hexagonal platelet shape corresponds to single crystalline Mg displaying prominent {0001} facets. The smaller NPs are more block-like but also appeared single crystalline by electron diffraction (Fig. S3, ESI†). This shape bimodality yielded a doubly peaked optical extinction spectrum (Fig. 1g), in contrast to the broad and flat spectrum of the heterogeneous Mg NPs synthesised using LiNapht only. Lastly, as expected, the reaction with the stronger reducing agent, Li_2_Napht, produced a higher reaction yield (29 ± 3%) than LiNapht (23.8 ± 1.1%).

The initial [Li_2_Napht] was controllable with sonication time (Fig. S4, ESI†) and measurable by a double titration.^32^ It significantly impacted the morphology of the Mg NPs produced in the first five minutes of the reaction. At 30% [Li_2_Napht], *i.e.*, when 30% of the naphthalene is in the dianion form and the remaining is a radical anion, large thin 470 ± 90 nm hexagonal platelets with concave indents were exclusively formed (Fig. 2a and Fig. S4, ESI†). They had a broad, flat optical extinction spectrum (Fig. 2e). At 49% and 75% [Li_2_Napht], small slightly rounded thick NPs with sizes of 56 ± 10 nm and 49 ± 12 nm, respectively, were formed (Fig. 2b–d and Fig. S5, ESI†). The formation of small NPs at higher [Li_2_Napht] is attributed to a faster reduction and increased nuclei density and is consistent with the trends observed for arene radical anions of increasing reduction potential.^19^ Optically, the NPs formed with 49% and 75% [Li_2_Napht] have well-defined extinction signatures peaking at ∼470 nm and 450 nm, respectively (Fig. 2e). The blue shift observed with smaller NPs is consistent with the peaks’ plasmonic origin, and the presence of such defined features indicates that single NPs are well-dispersed in the colloidal suspension. The loose aggregates observed in SEM are likely drying artifacts rather than growth aggregates, as also suggested by the presence of single NPs and sizeable gaps between aggregated NPs in both STEM and SEM.

**Fig. 2:**
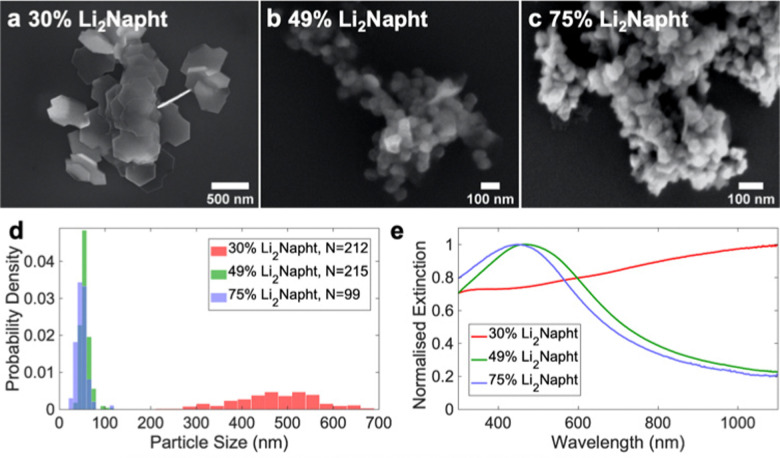
Effect of [Li_2_Napht] on the size and shape of Mg NPs produced after five minutes of reaction. (a–c) Representative SEM images, (d) size distributions and (e) optical extinction spectra of Mg NPs obtained with different [Li_2_Napht].

Looking back at the bimodal sample in view of the size and shape control provided by the [Li_2_Napht], we propose that the small NPs were first produced and, since the rate of reduction was fast, a kinetic shape was formed. The slower nucleation and growth of the large, closer to thermodynamic shape hexagonal platelets followed once the [Li_2_Napht] was depleted to ∼30%. This reaction mechanism is comparable to that of seeded growth syntheses of Au NPs,^21,22^ and, analogously to Au,^33^ the reducing agent dictates the prevalence of twinning. Here, ∼50% of NPs are twinned when using LiNapht and virtually none are observed with 30% or more Li_2_Napht.

Based on the hypothesis that a continuously changing concentration of dianion during the reaction leads to multiple shapes, we devised a two-step, seed-mediated approach for the synthesis of Mg NPs. A reaction of di-*n*-butylmagnesium with a 75% Li_2_Napht reducing agent solution was started, and, after five minutes, naphthalene in THF was added such that all the dianion was rapidly converted to LiNapht. The solution immediately turned from purple to dark green as previously reported,^26^ its reduction potential significantly weakened, and the reaction shifted from nucleation to predominantly growth. The seeds produced during the first five minutes in the Li_2_Napht-rich environment were ∼50 nm (Fig. 2c) and grew to thick faceted NPs with a mean size of 121 nm and a narrow size distribution (121 ± 9 nm; 7%) over 60 minutes after transforming the dianion to the radical anion (Fig. 3 and Fig. S6, ESI†). No further growth was observed after 60 minutes. The excellent dispersion of these structures provided further evidence of the lack of significant aggregation early in the reaction. The optical extinction spectrum displays a dipole and higher order plasmon resonances, as expected (Fig. 3d). An identical reaction, but without the additional naphthalene, yielded a faster reduction, as measured by ICP-MS (Table S1, ESI†), and a starkly different, bimodal shape and size distribution (Fig. 3a and c and Fig. S6, ESI†). These results highlight the effectiveness of separating the nucleation and growth stages *via* the *in situ* transformation of the arene dianion to the radical anion during the reaction.

**Fig. 3:**
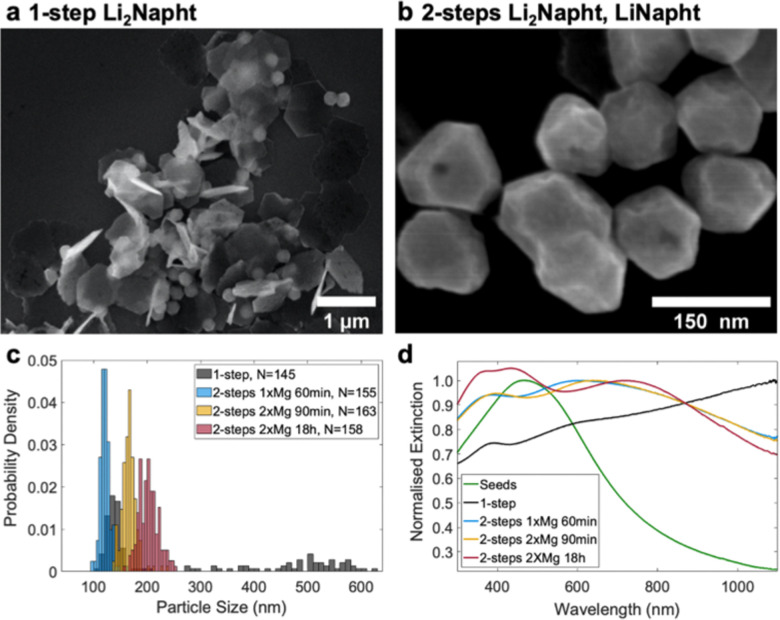
Comparison of one step and multiple seeded (two steps) Mg NPs syntheses. Representative SEM images for (a) one step 65 minutes reaction using Li_2_Napht and (b) two steps reaction starting with Li_2_Napht for five minutes followed by addition of sufficient naphthalene to convert all remaining Li_2_Napht to LiNapht. The reactions labelled 2xMg received additional Mg precursor 30 minutes after the naphthalene addition. (c) Size distributions for the single step and two steps reactions and (d) optical extinction spectra of Mg NPs synthesised in five minutes with Li_2_Napht (‘Seeds’), as well as the products of the one step and two steps reactions.

The precursor feed regime during the growth stage can be used to control NP size. Adding a second identical dose of Mg precursor 30 minutes after the naphthalene injection and letting the mixture to react for a further 60 minutes or 18h led to larger NPs of similar shapes, with monodisperse sizes of 166 ± 14 nm (8%) and 203 ± 18 nm (9%), respectively (Fig. 3c and d and Fig. S6, ESI†). The Mg NPs were confirmed to be metallic by STEM-EELS (Fig. S7, ESI†) and to be capped by a surface oxide by STEM-EDS (Fig. S8, ESI†). The observed continuous and uniform growth of Mg NPs rules out formation of an oxide layer during the synthesis as this layer would stop the growth of metallic Mg. Together with the highly reductive reaction medium this suggest that the oxide layer is formed during post-treatment. The plasmonic response of the increasingly large NPs shows a distinct redshift as expected (Fig. 3d).

The dianions of alternative arenes can also reduce organometallic Mg compounds. Biphenyl, phenanthrene and anthracene reacted with 2 equivalents of Li in THF under sonication for 45 minutes to produce green/blue Li_2_Biphenyl, brown/green Li_2_Phenanthrene, and deep purple Li_2_Anthracene solutions. After an 18h reaction with di-*n*-butylmagnesium, the strongest reducing agent, Li_2_Biphenyl, yielded the smallest NPs (46 ± 12 nm), followed in size by the product of the weaker Li_2_Phenanthrene (70 ± 20 nm), Fig. 4 and Fig. S9, ESI,† a trend consistent with results using their radical anion.^19^ As a comparison, the small NPs in the equivalent Li_2_Napht reaction were 54 ± 18 nm, in line with its reduction potential being in between that of Li_2_Biphenyl and Li_2_Phenanthrene. The weakest reducing agent, Li_2_Anthracene did not produce enough product for analysis.

**Fig. 4:**
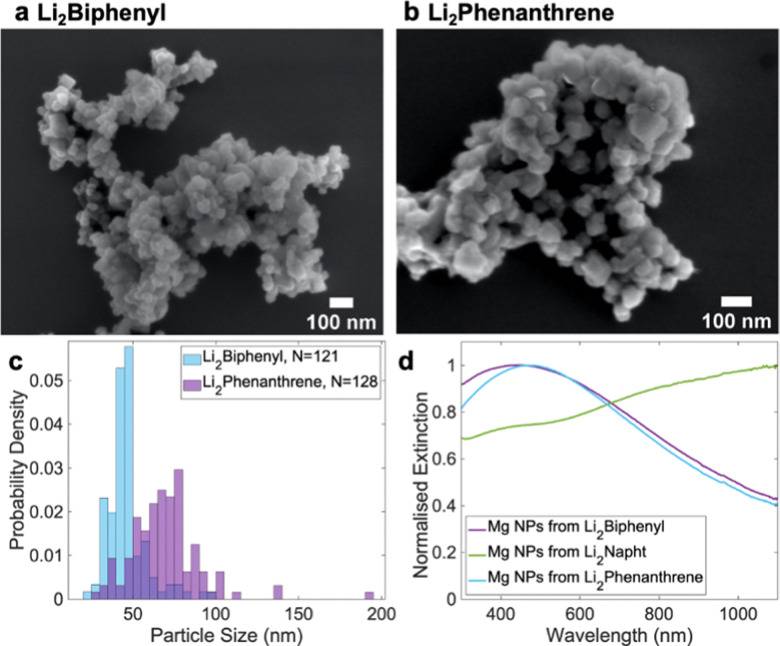
Effect of electron carrier on Mg NPs. (a and b) Representative SEM and (c) size distributions from reactions using Li_2_Biphenyl and Li_2_Phenanthrene, and (d) optical extinction spectra from reactions using Li_2_Biphenyl, Li_2_Napht, and Li_2_Phenanthrene.

Unlike with Li_2_Napht, the size distributions obtained with the other arenes were not bimodal, likely because the reaction does not span the reduction potential (and reduced Mg concentration) range required for the growth of two distinct populations. This narrow size distribution led to singly-peaked extinction spectra (Fig. 4d) with the expected redshift between small (Li_2_Biphenyl, peak at 430 nm) and large NPs (Li_2_Phenanthrene, 470 nm). The reaction yields also increased with reducing potential, from 11.3 ± 1.4% to 29 ± 3% to 44 ± 3% for Li_2_Phenanthrene, Li_2_Napht and Li_2_Biphenyl, respectively. There is thus a substantial array of reducing agents available for the synthesis of Mg NPs, and even more combinations possible in the seed-mediated, two-step approach described above.

In conclusion, we described the controlled production and use of arene dianions (Li_2_Biphenyl, Li_2_Naphthalene, and Li_2_Phenanthrene) for the reduction of di-*n*-butylmagnesium in the colloidal synthesis of metallic, plasmonic Mg NPs with improved size and shape dispersity. A novel two step, seed-mediated growth approach not previously reported for Mg was demonstrated. This reaction used a highly reductive Li arene dianion to induce the nucleation of single crystalline seeds; the reduction potential of the reaction medium was then modified rapidly by providing enough arene to produce only the radical anion. This first report of a successful seeded growth for Mg NPs paves the way for a new library of syntheses, yielding tuneable size with the monodispersity needed for the many promising applications of this earth-abundant, biocompatible plasmonic metal.

Support for this project was provided by the EU Framework Programme for Research and Innovation Horizon 2020 (ERC Starting Grant SPECs 804523) and the Engineering and Physical Science Research Council (EPSCR) grant EP/W015986/1 (MagNanoThermo) and EP/L015978/1 (Cambridge NanoDTC).

## Conflicts of interest

The authors are inventors on a pending patent application concerning the synthesis routes and nanoparticle shapes reported in this paper.

## Supplementary Material

CC-059-D3CC00958K-s001

## References

[cit1] Loo C., Lin A., Hirsch L., Lee M.-H., Barton J., Halas N., West J., Drezek R. (2004). Technol. Cancer Res. Treat..

[cit2] Baffou G., Quidant R. (2014). Chem. Soc. Rev..

[cit3] Christopher P., Xin H., Linic S. (2011). Nat. Chem..

[cit4] Gellé A., Moores A. (2019). Curr. Opin. Green Sustainable Chem..

[cit5] Mayer K. M., Hafner J. H. (2011). Chem. Rev..

[cit6] Langer J., Jimenez de Aberasturi D., Aizpurua J., Alvarez-Puebla R. A., Auguié B., Baumberg J. J., Bazan G. C., Bell S. E. J., Boisen A., Brolo A. G., Choo J., Cialla-May D., Deckert V., Fabris L., Faulds K., García de Abajo F. J., Goodacre R., Graham D., Haes A. J., Haynes C. L., Huck C., Itoh T., Käll M., Kneipp J., Kotov N. A., Kuang H., le Ru E. C., Lee H. K., Li J.-F., Ling X. Y., Maier S. A., Mayerhöfer T., Moskovits M., Murakoshi K., Nam J.-M., Nie S., Ozaki Y., Pastoriza-Santos I., Perez-Juste J., Popp J., Pucci A., Reich S., Ren B., Schatz G. C., Shegai T., Schlücker S., Tay L.-L., Thomas K. G., Tian Z.-Q., van Duyne R. P., Vo-Dinh T., Wang Y., Willets K. A., Xu C., Xu H., Xu Y., Yamamoto Y. S., Zhao B., Liz-Marzán L. M. (2020). ACS Nano.

[cit7] Fort E., Grésillon S. (2008). J. Phys. D: Appl. Phys..

[cit8] Salzemann C., Brioude A., Pileni M.-P. (2006). J. Phys. Chem. B.

[cit9] Gérard D., Gray S. K. (2014). J. Phys. D: Appl. Phys..

[cit10] Biggins J. S., Yazdi S., Ringe E. (2018). Nano Lett..

[cit11] Hopper E. R., Boukouvala C., Asselin J., Biggins J. S., Ringe E. (2022). J. Phys. Chem. C.

[cit12] Sterl F., Strohfeldt N., Walter R., Griessen R., Tittl A., Giessen H. (2015). Nano Lett..

[cit13] Ringe E. (2020). J. Phys. Chem. C.

[cit14] Jeong H.-H., Mark A. G., Fischer P. (2016). Chem. Commun..

[cit15] Martin R. C., Locatelli E., Li Y., Matteini P., Monaco I., Cui G., Li S., Banchelli M., Pini R., Comes Franchini M. (2016). J. Mater. Chem. B.

[cit16] Liu L., Wu Y., Ye J., Fu Q., Su L., Wu Z., Feng J., Chen Z., Song J. (2022). Chemistry.

[cit17] Lomonosov V., Wayman T. M. R., Hopper E. R., Ivanov Y. P., Divitini G., Ringe E. (2023). Nanoscale.

[cit18] Rieke R. D., Li P. T.-J., Burns T. P., Uhm S. T. (1981). J. Org. Chem..

[cit19] Hopper E. R., Wayman T. M. R., Asselin J., Pinho B., Boukouvala C., Torrente-Murciano L., Ringe E. (2022). J. Phys. Chem. C.

[cit20] Asselin J., Boukouvala C., Hopper E. R., Ramasse Q. M., Biggins J. S., Ringe E. (2020). ACS Nano.

[cit21] Xia Y., Gilroy K. D., Peng H.-C., Xia X. (2017). Angew. Chem., Int. Ed..

[cit22] Murphy C. J., Thompson L. B., Chernak D. J., Yang J. A., Sivapalan S. T., Boulos S. P., Huang J., Alkilany A. M., Sisco P. N. (2011). Curr. Opin. Colloid Interface Sci..

[cit23] Jacobson C. R., Solti D., Renard D., Yuan L., Lou M., Halas N. J. (2020). Acc. Chem. Res..

[cit24] Asselin J., Boukouvala C., Wu Y., Hopper E. R., Collins S. M., Biggins J. S., Ringe E. (2019). J. Chem. Phys..

[cit25] Asselin J., Hopper E. R., Ringe E. (2021). Nanoscale.

[cit26] Smid J. (1965). J. Am. Chem. Soc..

[cit27] Blasco I., Pérez H., Guijarro A. (2015). J. Phys. Org. Chem..

[cit28] Yus M., Herrera R. P., Guijarro A. (2001). Tetrahedron Lett..

[cit29] Liu W., Aguey-Zinsou K.-F. (2014). J. Mater. Chem. A.

[cit30] Song M.-R., Chen M., Zhang Z.-J. (2007). Mater. Charact..

[cit31] Jeon K.-J., Moon H. R., Ruminski A. M., Jiang B., Kisielowski C., Bardhan R., Urban J. J. (2011). Nat. Mater..

[cit32] Ager D. J., Organomet J. (1983). Chemistry.

[cit33] Zhang Q., Xie J., Yu Y., Yang J., Lee J. Y. (2010). Small.

