# Supplementary material for: Seed-mediated synthesis of monodisperse plasmonic magnesium nanoparticles
Source: Chem Commun (Camb). 2023 Apr 12;59(37):5603–6. doi: 10.1039/d3cc00958k (PMC10157803; doi:10.1039/d3cc00958k)
Supplement: CC-059-D3CC00958K-s001 [file CC-059-D3CC00958K-s001.pdf]

Electronic Supplementary Material (ESI) for ChemComm.
This journal is © The Royal Society of Chemistry 2023

Supplementary Information for

Seed-mediated synthesis of monodisperse plasmonic magnesium nanoparticles

Vladimir Lomonosov,[1,2,3] Elizabeth R. Hopper[1,2,3] & Emilie Ringe[1,2*]

1. Department of Materials Science and Metallurgy, University of Cambridge, 27 Charles Babbage Road, Cambridge, United Kingdom, CB3 0FS
2. Department of Earth Sciences, University of Cambridge, Downing Street, Cambridge, United Kingdom, CB2 3EQ
3. Department of Chemical Engineering and Biotechnology, University of Cambridge, Philippa Fawcett Drive, Cambridge, United Kingdom, CB3 0AS

* Corresponding author: er407@cam.ac.uk; +44 (0)1223 334330 (ph.)

Methods

Materials

Li pellets (99%), naphthalene, biphenyl, phenanthrene, anthracene, 1.0 M di-*n*-butylmagnesium ($MgBu_2$) in heptane, poly(vinyl pyrrolidone) (PVP, M_W 10,000), phenolphthalein, ethylene bromide, hydrochloric acid, anhydrous tetrahydrofuran (THF) and anhydrous isopropanol (IPA) were purchased from Sigma-Aldrich and used as supplied. All glassware was washed with nitric acid and flame-dried under vacuum before use.

Mg NP synthesis

General effect of arene dianions on Mg synthesis.

To investigate the general effect of the dianion on colloidal Mg synthesis, Mg NPs were synthesised from an organometallic precursor, di-n-butylmagnesium ($MgBu_2$), using the Li_2Napht dianion or the LiNapht radical anion as the reducing agent. A typical procedure for Li_2Napht formation was as follows: 0.028 g of Li pellets (4.05 mmol), 0.260 g of dry naphthalene (2.03 mmol), 20 mg PVP (0.18 mmol monomer) and 10.75 mL of degassed anhydrous THF were added to a 25 mL Schlenk flask under Ar atmosphere and sonicated for 45 minutes (Allendale Ultrasonics, 100 W 3 L). LiNapht was prepared following the same protocol using twice the amount of naphthalene (0.530 g, 4.05 mmol). The effect of the electron carrier was investigated using biphenyl, phenanthrene and anthracene as an alternative to naphthalene. In a typical synthesis of Mg NPs, 1.75 mL of $MgBu_2$ in heptane (1.0 M) was injected quickly into freshly prepared reducing agent and the reaction was allowed to proceed for 18 hours at room temperature (20 ^oC) before quenching with 2 mL of anhydrous IPA. The solid product was recovered by centrifugation and residual by-products were removed by centrifugation and redispersion steps in anhydrous IPA twice, anhydrous THF twice and anhydrous IPA twice, before redispersing in anhydrous IPA.

WARNING: $MgBu_2$ is pyrophoric and should be handled and transferred in air-free conditions.

Effect of Li_2Napht concentration.

In this series of experiments, Li_2Napht was prepared as described above, but the sonication time was varied from 15 to 60 minutes to obtain different Li_2Napht/LiNapht ratios in the resulting reducing agent.

The concentration of Li_2Napht increased with sonication time and reached its highest level of 75% of the theoretical maximum after 45 minutes, being 30% after 15 minutes (when the purple colour had

just started to form), 49% after 30 minutes and 59% after 1 hour (Figure S4). The longer sonication time leads to a decrease in the dianion concentration apparently due to its reaction with THF, which can be facilitated under sonication and by rising temperatures in the sonication bath.

To elucidate the effect of the Li_2Napht concentration on the initial stages of Mg NP formation, the reaction between the reducing agent and $MgBu_2$ was quenched after 5 minutes with 2 mL of anhydrous IPA.

Seed mediated synthesis of Mg NPs

In these experiments, the reduction potential of the reaction mixture was changed *in-situ* by transforming Li_2Napht to LiNapht with an excess of naphthalene. Mg NP synthesis was initiated by injecting quickly 1.75 mL of $MgBu_2$ in heptane (1.0 M) into freshly prepared Li_2Napht solution followed by addition of 2 mL of naphthalene in THF (1.0 M) after 5 minutes of reaction, to convert all unreacted Li_2Napht to LiNapht. The resulting mixture was left to react for 60 minutes and then quenched with 2 mL of IPA and cleaned as described above. This reaction is called 2-steps 1xMg 60 min. A further dose of 1.75 mL of $MgBu_2$ in heptane (1.0 M) was added 30 minutes after the naphthalene addition and left to react either a further 60 minutes (2-steps 2xMg 90 min) or 18 hours (2-steps 2xMg 18h).

Characterisation

Double titration method

The amount of Li_2Napht dianion in the reduction solution was determined by a double titration method.[27,28] To find the total amount of Li present, a 0.5 mL aliquot of the dianion solution was hydrolysed with 5 mL of deionised water and titrated against standardised 0.1 M hydrochloric acid with phenolphthalein as an indicator. The amount of residual (non-reactive) Li was then found by quenching another 0.5 mL of dianion solution with 1 mL of ethylene bromide and titrating against the hydrochloric acid after dilution with 5 mL water. The amount of Li_2Napht was calculated based on the difference between the amount of reactive Li determined by double titration and the amount of naphthalene used to prepare the dianion solution.

Characterisation of Mg NPs

Freshly prepared Mg NP samples were drop cast onto Si wafers for scanning electron microscopy (SEM) imaging, performed on a FEI Nova NanoSEM, operated at 5 kV and equipped with an in-lens detector for secondary electron imaging. High-angle annular dark field scanning transmission electron microscopy (HAADF-STEM) images, electron diffraction patterns, STEM energy dispersive X-ray spectroscopy (STEM-EDS) maps and STEM electron energy loss spectroscopy (STEM-EELS) maps

of samples drop cast on a Cu-supported lacey ultrathin carbon membrane were acquired at 200 kV on a FEI Osiris STEM with a Bruker Super-X quadruple EDS detector and a Gatan Enfinium ER 977 electron spectrometer, except for the HAADF-STEM, STEM-EDS and STEM-EELS shown in Figures S6-S7 which were acquired on a Thermo Fisher Spectra equipped with a Bruker Super-X quadruple EDS detector and a Gatan Continuum electron spectrometer. STEM-EELS maps of the Mg bulk plasmon (Fig. 1h, S2) were produced by integrating the intensity of the spectrum image from 9.5 to 11.5 eV, and STEM-EDS maps (Fig. 1f) were obtained by integrating the Kα peaks of O and Mg.

NP size distributions were obtained by measuring at least 90 NPs with clearly visible edges from SEM images. The sizes measured were the longest dimension, *e.g.*, the distance between opposite corners for a hexagonal platelet or the longest length visible of thick, block-like NPs. Reported size polydispersities are the standard deviation of all measurements for monomodal samples, and the standard deviation of measurements of each shape type for bimodal samples.

Inductively coupled plasma mass spectrometry (ICP-MS) was performed using a Perkin Elmer Nexion 350D quadrupole-based mass spectrometer. Samples were digested in a 0.2 M nitric acid matrix and diluted to around 10 ppb. Errors on the ICP-MS measurements are reported as the standard deviation of three measurements of the same sample. Extinction spectra were measured using a Thermo Scientific Evolution 220 spectrophotometer.

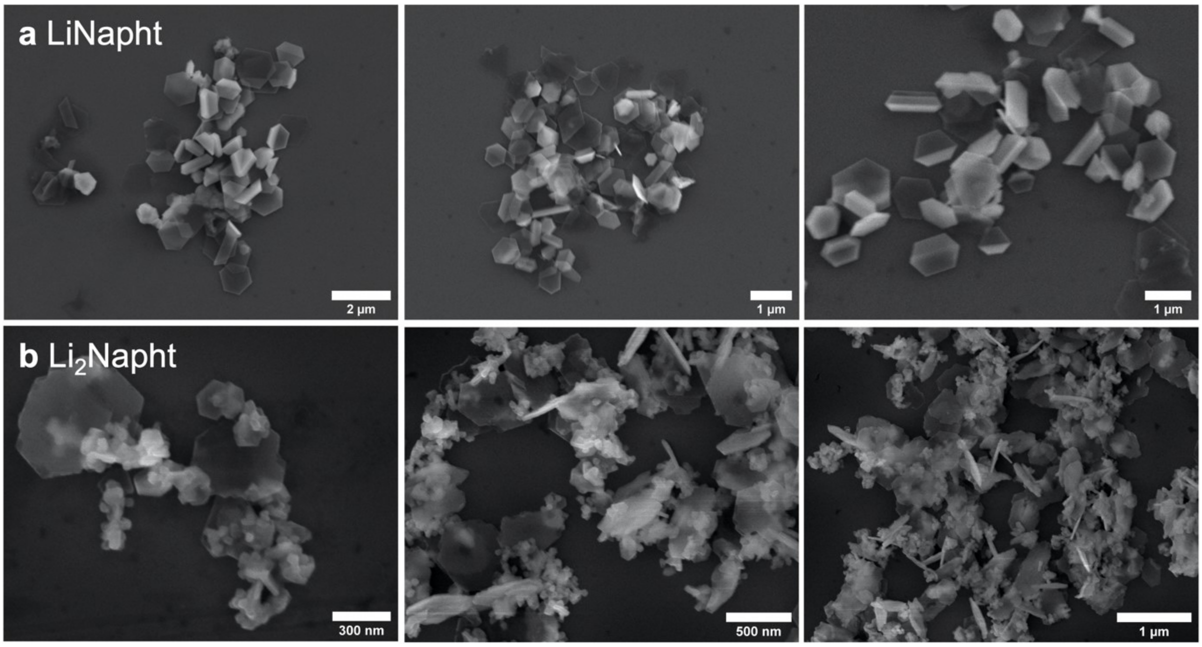

Figure S1. Additional representative SEM images of Mg NPs synthesised using (a) 1:1 Li:Napht (LiNapht) and (b) 2:1 Li:Napht (Li_2Napht), respectively.

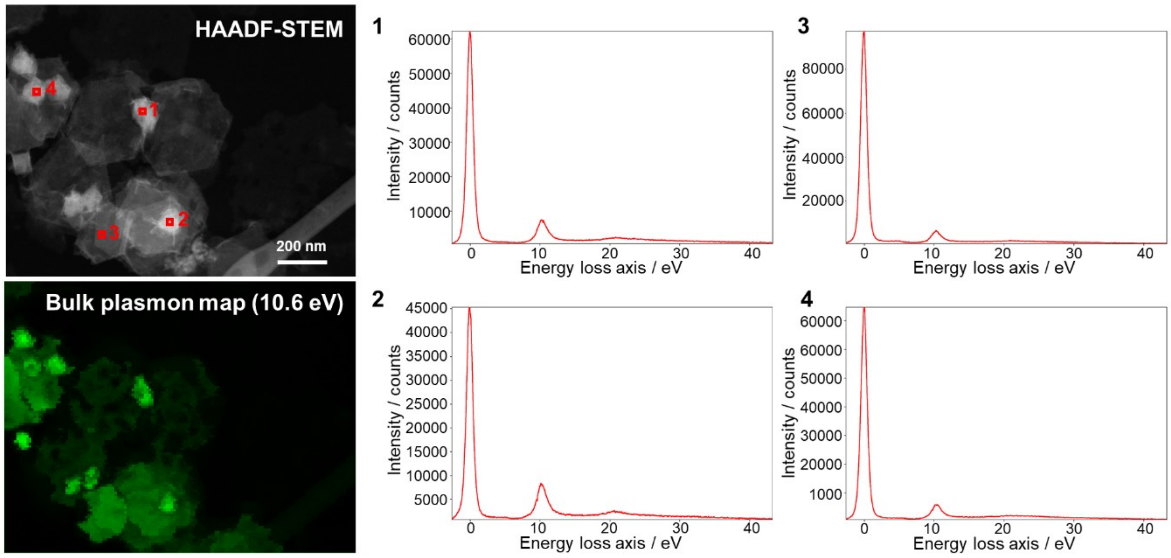

Figure S2. Full HAADF-STEM image and STEM-EELS map from Fig. 1, and STEM-EELS spectra from the positions labelled showing the ~10.6 eV bulk plasmon peak of Mg.

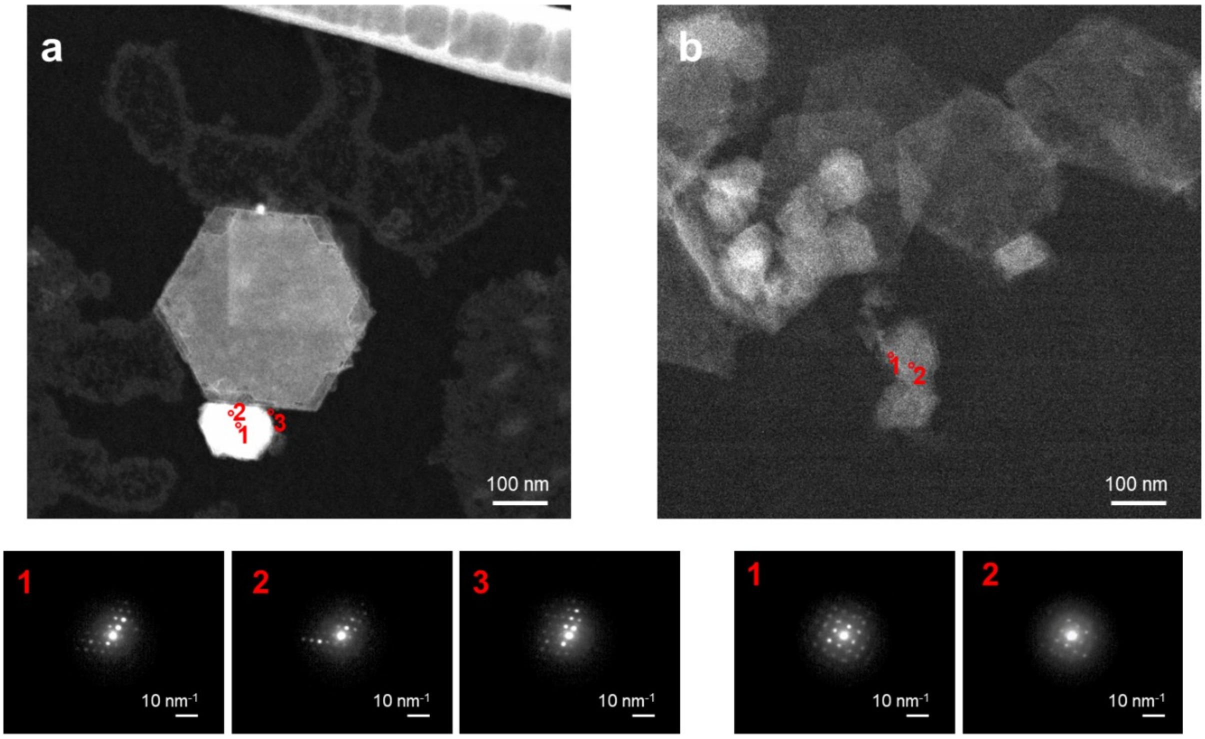

Figure S3. Crystallinity of small Mg NPs from a bimodal sample: diffraction patterns at several positions on the same NP showing no change in the pattern symmetry.

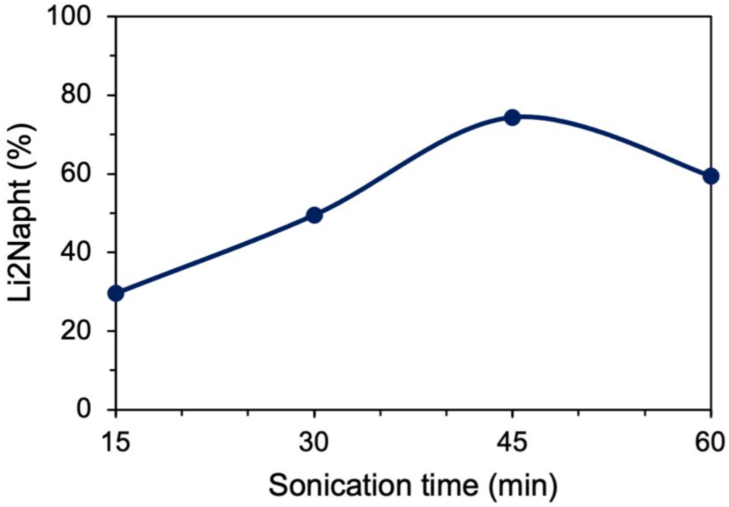

Fig. S4. [Li_2Napht] produced from a 2:1 Li:Napht mixture in THF as a function of sonication time. The [Li_2Napht] is reported such that 30% [Li_2Napht] means that 30% of the total naphthalene is in the dianion form and the remaining 70% is a radical anion.

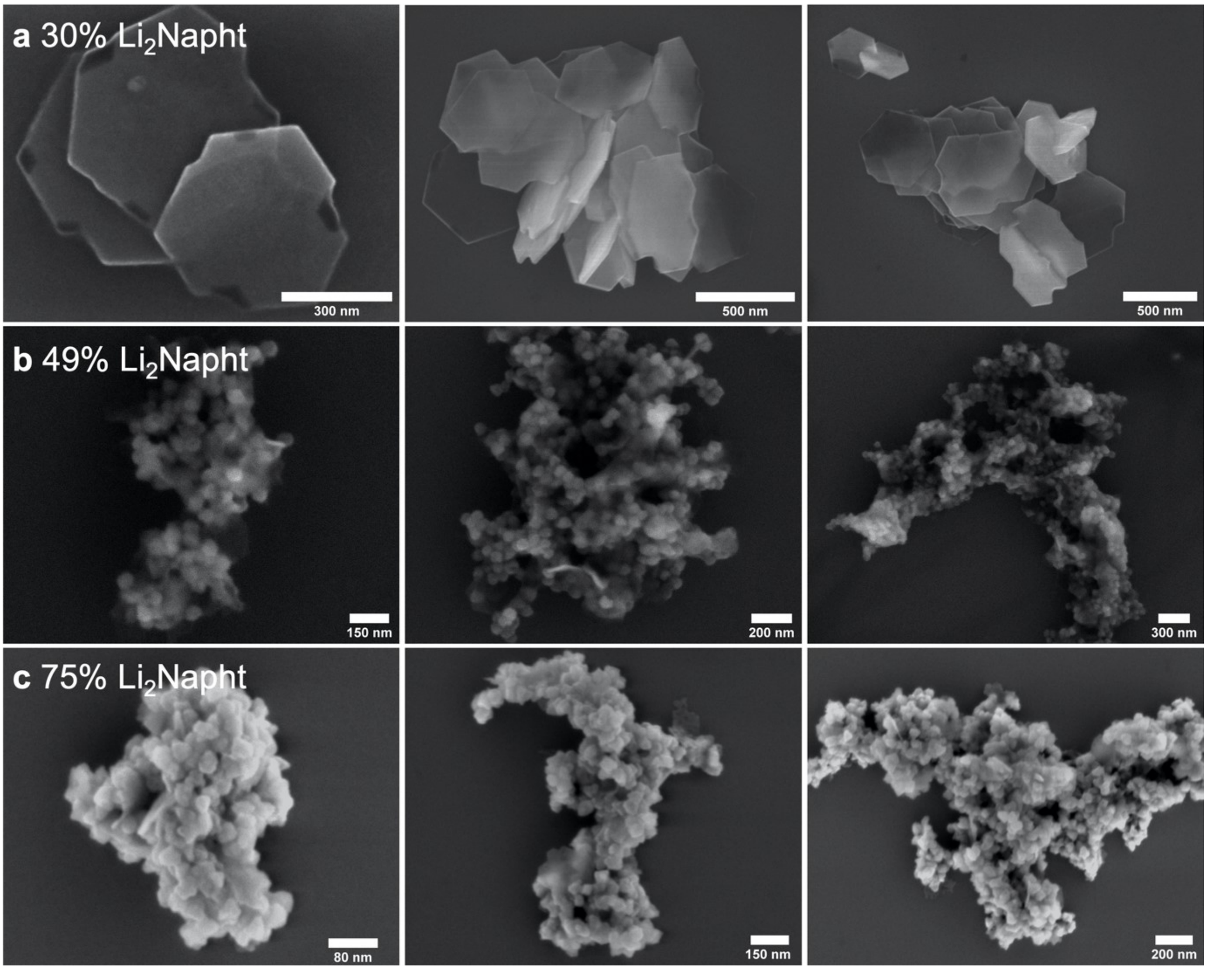

Figure S5. Additional SEM images supporting Fig. 2 on the effect of Li_2Napht concentration on Mg NPs.

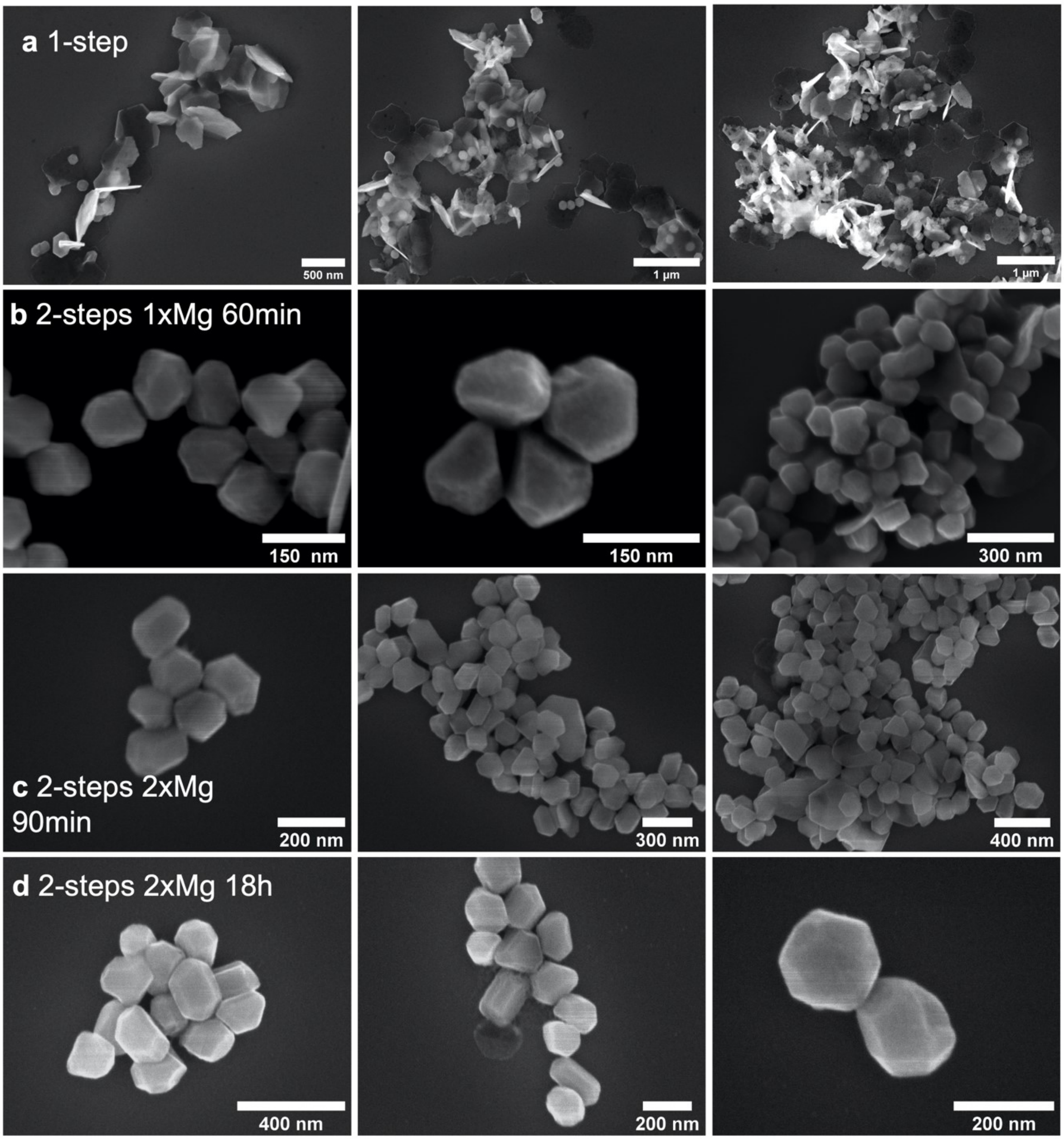

Figure S6. Additional SEM images supporting Fig. 3 on the effect of a two-step seeded growth and on the addition, during the growth stage, of additional Mg precursor.

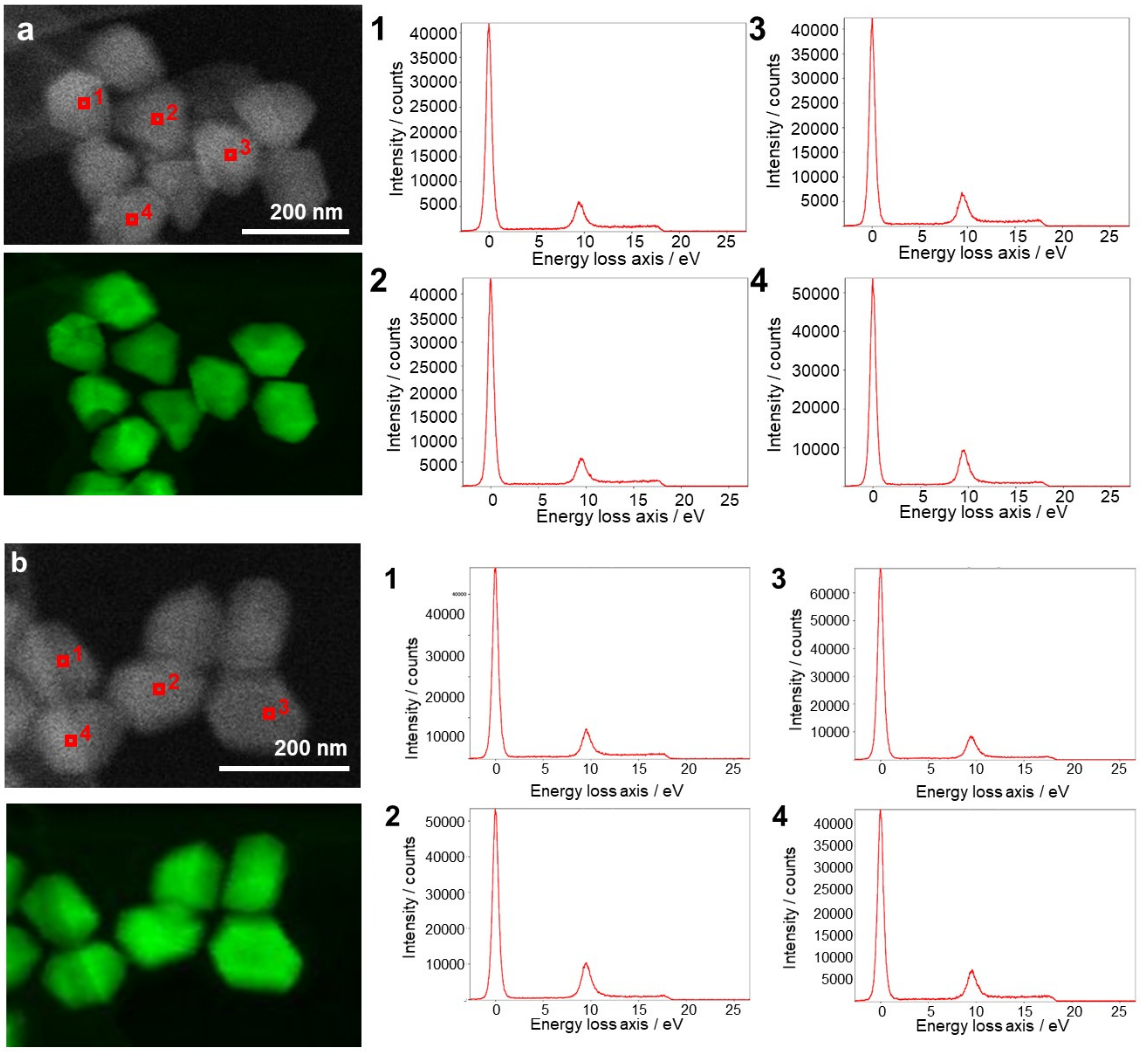

Figure S7. Metallic character of Mg NPs from the 2 steps 2xMg 90 minutes seeded growth reaction confirmed by STEM-EELS. For (a) and (b), HAADF-STEM images, maps of the bulk plasmon of Mg at 10.6 eV and (1)-(4) EELS spectra at the points marked in the HAADF-STEM images with a red box.

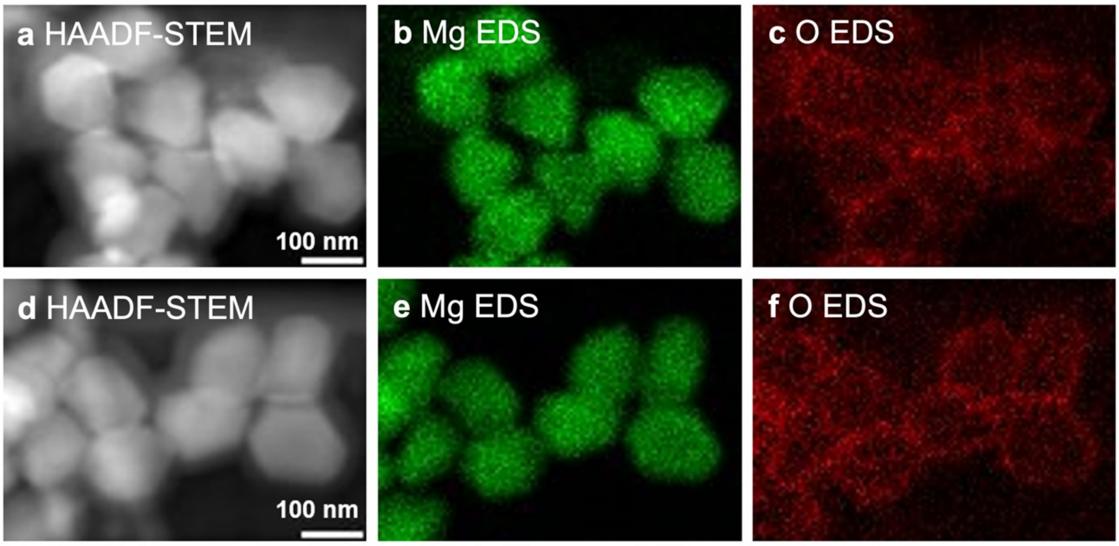

Figure S8. Composition of Mg NPs Mg NPs from the 2 steps 2xMg 90 minutes seeded growth reaction: (a, d) STEM-HAADF, and intensity maps of (b, e) Mg Kα, and (c, f) O Kα measured by STEM-EDS.

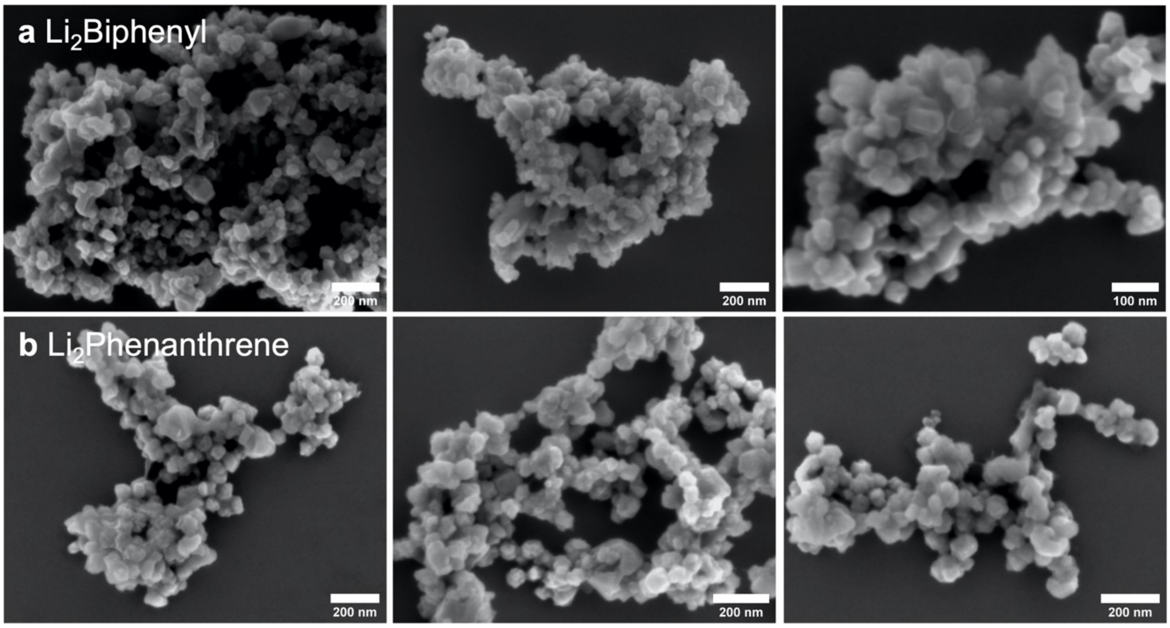

Figure S9. Additional SEM images supporting Fig. 4 on the effect of the electron carrier on Mg NPs. Reduction with (a) Li_2Biphenyl and (b) Li_2Phenanthrene.

Table S1. Amount of product and reaction yield as measured by ICP-MS.

**Reaction label**	**Amount of product / mg**	**Yield / %**
LiNapht 18 hours (Fig. 1)	10.1 ± 0.5	23.8 ± 1.1
Li_2Napht 18 hours (Fig. 1, 4)	12.3 ± 1.1	29 ± 3
75% Li_2Napht 5 min (seeds) (Fig. 2, 3)	0.70 ± 0.15	1.7 ± 0.4
75% Li_2Napht 1 hour (control) (Fig. 3)	8.9 ± 0.7	21.0 ± 1.7
Li_2Biphenyl 18 hours (Fig. 4)	18.8 ± 1.2	44 ± 3
Li_2Phenanthrene 18 hours (Fig. 4)	4.8 ± 0.6	11.3 ± 1.4
